# Editorial: Pest-smart strategies for improved eco-efficiency in agriculture, forestry and communities

**DOI:** 10.3389/finsc.2026.1795406

**Published:** 2026-03-03

**Authors:** George B. Frisvold, Thomas M. Chappell, Ashfaq A. Sial, Roger D. Magarey

**Affiliations:** 1 Department of Agricultural and Applied Economics, University of Arizona, Tucson, AZ, United States; 2 Department of Plant Pathology and Microbiology, Texas A&M University, College Station, TX, United States; 3 Institute for Integrative Precision Agriculture, University of Georgia, Athens, GA, United States; 4 Department of Entomology, University of Georgia, Athens, GA, United States; 5 Center for Integrated Pest Management, North Carolina State University, Raleigh, NC, United States

**Keywords:** Eco-efficiency, externalities, FIFRA, IPM, performance standard

## 1 Limits of integrated pest management implementation

The concept of economic thresholds in IPM (the pest density where control measures should be implemented to prevent economic injury) begins with a powerful argument: the cost of pesticide applications should not exceed the economic losses the applications prevent. Under IPM, knowledge of agronomic and biological systems substitutes for chemicals, increasing farm income. This can produce external benefits, as pesticides have ecological and human health costs. IPM can be a “win-win” strategy: improving farming productivity and profitability, while reducing environmental damage. Yet the U.S. General Accounting Office found problems with federal IPM initiatives (1). Despite a 70% IPM adoption rate on U.S. crop acreage, “USDA counts a wide variety of farming practices without distinguishing between those that tend to reduce chemical pesticide use from those that may not” and “USDA and EPA suggested that an appropriate objective for IPM could be reduction in pesticide risk to human health and the environment, but neither agency adopted that objective.” Further, “IPM [ … ] has not yet yielded nationwide reductions in chemical pesticide use.”

What went wrong? USDA and EPA were aware that the pertinent issue was *harm*, not the physical quantity of pesticides used. But harm (such as acute toxicity, chronic health effects, biodiversity loss, water contamination, or resistance development) is harder to predict and measure. Instead, IPM was treated as a technology standard, not a performance standard (2–4), with progress measured in terms of practice adoption. But linkages between adoption and environmental outcomes can be tenuous. The case of water conservation illustrates. The policy consensus is that improving efficiency conserves water, with progress measured in terms of adopting “efficient” irrigation (5, 6). Yet, the scientific consensus is that, under most conditions, improving irrigation efficiency increases water consumption (5, 6). A performance goal (reducing harm from pesticides) would allow producers to achieve that goal in the most cost-effective manner. With a goal of prescribed practice adoption, there is no incentive to innovate to reduce harm. Also, voluntary adoption relies on farmers weighing private costs and benefits. There is no reason such private calculations would address external costs of pesticide use.

## 2 Limits of current regulatory framework

The Federal Insecticide, Fungicide, and Rodenticide Act (FIFRA) requires the U.S. Environmental Protection Agency (EPA) to evaluate risks and benefits of pesticides before they are registered for use. Pesticides must not cause “unreasonable adverse effects on the environment” defined as unreasonable risk to humans or the environment, considering economic, social, and environmental costs and benefits, and unacceptable dietary risk from pesticide residues (7). EPA conducts risk-benefit evaluations. Risks are measured in terms of human health (e.g., toxicity and exposure) and environmental damage (e.g., toxicity to non-target species and ecosystem harm). Benefits to pesticide users are measured in terms of crop yield and quality, and farm costs and returns. This system attempts to quantify tradeoffs between agricultural productivity and profitability and ecological and health risks in pesticide use.

There are limits to how well the FIFRA framework balances these tradeoffs. It is a framework for managing pesticide use, not one for managing pests. It establishes a minimum standard of environmental protection for how and where pesticides can be used with limited consideration of non-chemical options. Because it sets minimum standards, it doesn’t encourage additional innovation to reduce harm further. The framework has difficulties managing risks *ex-post*. Risks are often unanticipated (e.g., resistance to glyphosate (8, 9), effects on pollinators (10, 11), or cancer risks (12, 13), manifesting themselves with lags. Substituting away from a compound becomes costly once it is embedded in farming systems. The process is litigious and adversarial, and subject to “regulatory capture” where regulator behavior can be (unduly) influenced by regulated industries (14).

## 3 Eco-efficiency: toward measuring and improving performance

The USDA National Roadmap for IPM shifted toward assessing performance, not just practice adoption (15). It recommended cost-benefit analysis, including external environmental and health costs. Economists have developed non-market valuation techniques one, in theory, could apply to such analysis. A report to EPA’s Pesticide Program Dialogue Committee, however, found, “It is not feasible [ … ] for EPA to conduct such analyses for every pesticide (or even large numbers of them),” recommending developing a priority system to evaluate a subset of compounds (16).

Eco-efficiency measures have been proposed to quantify economic and environmental performance of pest management in a single index: a ratio of agricultural output to a measure of potential for environmental harm (based on quantity and toxicity of pesticides used) (Kreick et al., Love et al., 17). Such indexes measure outcomes, not practices. They may inform environmental certification programs to internalize externalities, allowing farmers to capture higher prices for environmentally friendly practices (17). Index values can be developed for major production systems across wide areas, accounting for most pesticide use (Love et al.).

We propose research opportunities concerning eco-efficiency and IPM.

- Determine under what circumstances increases in eco-efficiency indexes translate into reductions in environmental harm, avoiding the “ratio trap.” There are myriad examples where improved efficiency ratios correlate poorly, or even negatively, with environmental goals (5, 6, 18–22). Examples date back to the Jevons paradox (20).
- Refine quantitative indices of costs to be minimized (Love et al.), with indices specific to use cases and community priorities; broader scope may not translate into better utility.
- Integrate indices to optimize system-level targets (Tiffin et al.), enabling comparisons of approaches that may all qualitatively represent One Health, but which differ in overall merit.
- Improve methods for observing pest and pathogen subsystems (Defilippo et al., Yanchenko et al.). Measure more than what is easy to measure (e.g., natural enemy population densities). This requires increasing the efficiency of observation.
- Integrate results from the above opportunities into frameworks that can be enhanced by data science and/or artificial intelligence (AI).

**Figure 1:**
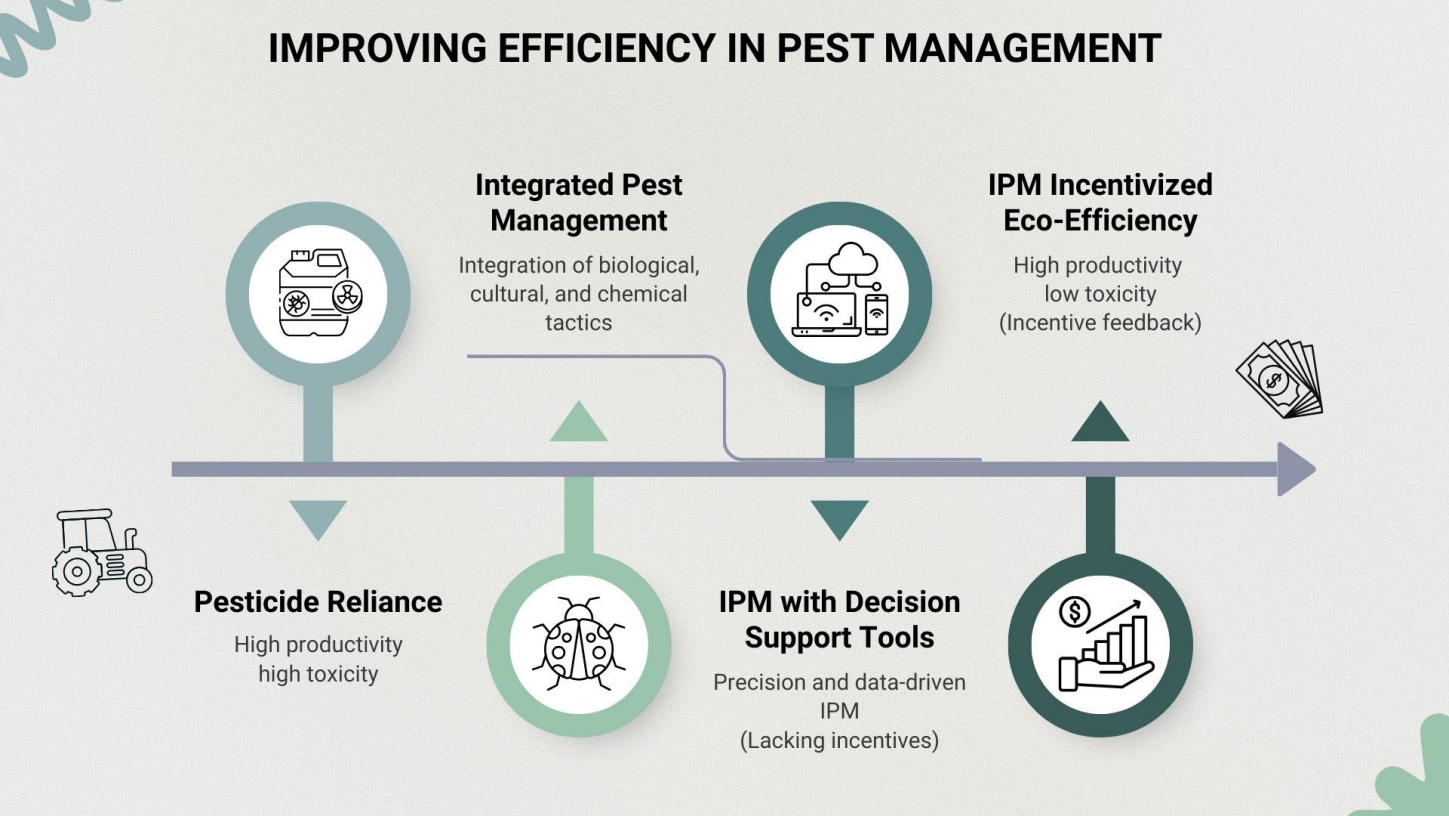
Conceptual progression toward incentive-aligned IPM systems that increase eco-efficiency: the ratio of productivity to negative impacts.

Eco-efficiency can be a critical part of a Pest-Smart Agriculture strategy (analogous to Climate-Smart Agriculture) to communicate, identify, quantify, track, and incentivize (via market price premiums, for example) ecologically friendly pest management. Eco-efficiency is not a replacement for IPM, but a means to better communicate the impacts and benefits of IPM (**Figure 1**). Advances in AI and data science can improve observation of pest and natural enemy systems, integrating diverse cost and benefit metrics, supporting longitudinal evaluation of performance. Eco-efficiency can improve pest management at multiple scales. On the scale of individual fields, eco-efficiency metrics can help guide farmer decision-making. At state, regional, or national levels, they can inform research, regulatory, and extension priorities, and support investments to reduce the environmental and health costs of pest management. By providing measurable metrics, Pest-Smart Agriculture could address the concerns outlined by the GAO (1) regarding the lack of metrics for evaluating economic and environmental outcomes of IPM. It could also better reflect the USDA IPM Roadmap’s call for greater emphasis on performance and more comprehensive measurement of that performance (15).
